# Nutrigenomics and Epigenetics in the Dietary Management of Inflammatory Bowel Diseases

**DOI:** 10.3390/genes16111368

**Published:** 2025-11-11

**Authors:** Patrycja Musz, Gabriela Ryś, Weronika Fic, Aneta Sokal-Dembowska, Sara Jarmakiewicz-Czaja

**Affiliations:** 1 Faculty of Health Sciences and Psychology, Collegium Medicum, University of Rzeszow, 35-959 Rzeszow, Poland; 2 Student Scientific Club of Human Nutrition, Faculty of Health Sciences and Psychology, Collegium Medicum, University of Rzeszow, ul. Warzywna 1a, 35-959 Rzeszow, Poland

**Keywords:** Crohn’s disease, ulcerative colitis, inflammatory bowel disease, diet, epigenetics

## Abstract

Inflammatory bowel diseases (IBD), including Crohn’s disease (CD) and ulcerative colitis (UC), are chronic diseases with complex aetiology involving genetic, immunological, and environmental factors and intestinal microbiota disorders. Mutations in genes such as NOD2, ATG16L1, IRGM, TLR4, and IL23R disrupt the functioning of the intestinal barrier and the immune response, increasing susceptibility to chronic inflammation. Recent studies indicate that interactions between diet, gene expression, and epigenetic mechanisms play a key role in modulating the course of IBD, e.g., DNA methylation, histone modifications, and microRNA activity. The use of bioactive dietary components in combination with epigenome modulation is a promising tool in the treatment of IBD, enabling the reduction in chronic inflammation, improving intestinal barrier function, and supporting the immune response.

## 1. Introduction

Inflammatory bowel diseases are chronic diseases. The two main diseases that belong to IBD are Crohn’s disease (CD) and ulcerative colitis (UC). Inflammation in CD can occur throughout the entire digestive tract and is separated by healthy sections. In UC, there is continuous inflammation, which is located in the large intestine and/or rectum. Diseases progress with periods of exacerbation and remission, and etiopathogenesis is multifactorial, i.e., environmental, immunological, and genetic factors and intestinal microbiota disorders [1,2]. Among environmental factors, lifestyle plays an important role, e.g., diet, physical activity, and use of stimulants. Sun et al. show in their study that an appropriate diet, free of ultra-processed foods, and an adequate level of physical activity may be independently associated with susceptibility to IBD [3]. IBD is a burden on healthcare systems around the world due to the increase in the number of patients [4,5]. Western countries are in the accumulation phase of the incidence of IBD, while developing countries are in the acceleration phase [6]. That is why researchers are looking for new solutions in therapy and prevention. Increasingly, attention is being drawn to nutrigenomics, the science that describes the relationship between individual food components and gene expression. Understanding how certain bioactive compounds, such as fatty acids, polyphenols, or vitamins, can modulate molecular pathways associated with the inflammatory process offers certain therapeutic possibilities in IBD [6,7]. Another equally important factor is epigenetics. Epigenetic mechanisms can supervise and regulate many pathophysiological and physiological processes. Environmental factors can cause reversible epigenetic changes [8]. The purpose of this article is to present current knowledge on the role of nutrigenomics and epigenetics in IBD and to discuss the mechanisms and therapeutic potential of interactions between genes, diet and the epigenome.

## 2. Methods

The search for studies forming the basis of the article took place between July and September 2025 and mainly included full studies in English published in PubMed, ScienceDirect, and Google Scholar. An electronic search strategy involving MeSH and keywords was used. The search strategy used a combination of relevant keywords and phrases: “Epigenetics,” “DNA Methylation,” “Histone Modifications,” “microRNA,” “Inflammatory Bowel Diseases,” “IBD,” “Crohn’s Disease,” “Ulcerative Colitis,” “Diet,” “Nutrition,” “Polyphenols,” “Omega-3”, “Short Chain Fatty Acids”, ‘Folate’ OR “Vitamin D”.

## 3. Genetic Determinants of Inflammatory Bowel Disease and Their Modification Through Diet

### 3.1. Selected Genes Associated with IBD

Key genes involved in IBD pathogenesis, including NOD2, ATG16L1 and IL23R, regulate innate immunity, autophagy, and T-cell differentiation, providing potential molecular targets for intervention [9,10,11]. The mucous membrane of the digestive tract acts as an active barrier protecting the body against the penetration of harmful microorganisms and toxins, while at the same time enabling the absorption of nutrients [12]. When crossing the intestinal barrier, microorganisms stimulate the immune system, leading to the secretion of a number of pro-inflammatory and anti-inflammatory mediators [13]. The proper functioning of genes involved in the functioning of the intestinal barrier ensures its proper efficiency and stability. In the event of disorders of the mechanisms or mutations of any of the genes, the intestinal defence line may be disrupted and the body’s homeostasis disturbed, which promotes the development of IBD [9,13]. Polymorphism of the NOD2 gene (nucleotide-binding oligomerisation domain-containing protein 2) plays an important role in the pathogenesis of CD, being one of the risk factors [14,15,16]. It is responsible for encoding a protein that acts as a receptor, identifying components of the wall of pathogenic bacteria and triggering the process of autophagy [17]. When activated by MDP (muramyl dipeptide), NOD2 activates a cascade of signals within the cell, which regulates the mechanism of the immune response of the body, thus maintaining the balance between the gut microbiota and the immune system. Mutations in the NOD2 gene disrupt these mechanisms, causing chronic inflammation. Lower NOD2 activity results in a weaker response to bacterial pathogens and, in some cases, can completely inhibit the body’s response. This can lead to increased susceptibility to inflammatory diseases but also to gastrointestinal cancers [14,16]. ATG16L1 (autophagy-related 16-like 1 protein) is an important component of the autophagy process, particularly within intestinal epithelial cells. Its task is to break down unnecessary and damaged products and then use them to produce energy and new proteins. The autophagy process also extends to pathogens, which are ultimately degraded. Mutations in the ATG16L1 gene disrupt this process, leading to a reduced ability of the body to fight bacteria and thus increasing the risk of developing intestinal diseases by altering intestinal barrier homeostasis. In addition, it can cause an increase in interleukin production (IL-1β), which in turn exacerbates inflammation in CD [18,19]. IRGM (immunity-related guanosine triphosphate M), on the other hand, is responsible for the elimination of intracellular pathogens. Like ATG16L1, it helps cells remove bacteria through autophagy. When this gene is mutated, cells are less effective in removing bacteria, which promotes the development of inflammatory diseases and damage to the intestinal barrier [18,20]. Furthermore, IRGM dysfunction can cause abnormalities in Paneth cells, which are involved in intestinal defence responses. Consequently, this may lead to increased susceptibility to IBD, but further research is needed in this area [21]. TLR receptors belong to PRRs (pattern recognition receptors), which are present in various cells of the immune system. After recognising microorganisms, TLR receptors undergo dimerisation and recruit adapter proteins that activate transcription factors, including NF-κB and AP-1, leading to the production of inflammatory mediators [22]. Excessive activation of the TLR4 gene, which encodes a transmembrane protein, can damage the structure of the intestinal mucosa and destroy stem cells and mucus-secreting goblet cells, which directly translates into disturbances in the integrity of the intestinal barrier [11]. Excess TLR4 stimulation also reduces the number of gut bacteria that produce SCFAs (short-chain fatty acids), which further promotes the development of IBD [22]. IL23R (Interleukin 23 receptor) also plays an important role in the development of chronic intestinal inflammation, among other things by regulating the immune response associated with Th17 lymphocyte activity. IL-23R mutations increase Th17 activity, leading to an increased inflammatory response and an increased production of pro-inflammatory cytokines. Chronic activation of the IL-23-Th17 axis can damage the intestinal mucosa and have a negative impact on the functioning of the intestinal barrier. An increase in the number of Th17 cells will also affect collagen deposition in the colon, leading to pathological intestinal fibrosis [16,18,19,23]. In addition, IL-23 signalling can inhibit regulatory T cell differentiation, which counteracts the onset of inflammation [24].

### 3.2. The Potential of Dietary Components to Modulate Gene Expression

Eating habits and the type of food products chosen are extremely important for the proper functioning of the human body, especially in the case of chronic diseases [25]. A Western-style diet, rich in fats and sugar, has been linked to IBD pathogenesis through modulation of microbiota and inflammatory responses [26]. In the context of IBD treatment, nutrigenomics provides a better understanding of how specific food components can modulate the inflammatory response and affect the integrity of the intestinal barrier, which gives a broader view of the development of personalised nutritional therapies [27]. Omega-3 fatty acids (docosahexaenoic acid (DHA) and eicosapentaenoic acid (EPA)) are polyunsaturated fatty acids with anti-inflammatory and immunomodulatory properties [13]. Their main sources include fatty sea fish, vegetable oils, nuts, and flaxseed [28]. Yulan et al. indicate in their study that supplementation with omega-3-rich fish oil may contribute to maintaining the integrity of the intestinal barrier. This may be related to its inhibitory effect on the expression of pro-inflammatory cytokines associated with the modulation of the inflammatory response linked to TLR4 and NOD2 [29]. Some studies suggest that omega-3 fatty acids may influence epigenetic processes associated with inflammation. This results in increased expression of anti-inflammatory genes and a simultaneous decrease in the activity of pro-inflammatory genes [30]. In addition, the EPA- and DHA-activated fatty acid receptor GPR120 inhibits the pathway that leads to the activation of NF-κB, which in turn reduces the transcription of pro-inflammatory genes in intestinal epithelial cells and macrophages [31]. As a result of fermentation in the large intestine, the fibre is converted to SCFA, which is responsible mainly for anti-inflammatory effects [32]. SCFAs are compounds that include acetate, butyrate, and propionate, which are very important in the regulation of inflammatory responses. A diet rich in dietary fibre will increase the production of SCFAs, which also affect the nutrition of the intestinal epithelium, the modulation of the intestinal microbiome, and the functioning of the immune system [22,30,33,34]. The research focused on the action of SCFA, mainly butyrate, and emphasises its special role in the regulation of the immune response in IBD. By acting on the GPR43 and GPR109a receptors, butyrate effectively limits the development of the disease, and its intake leads to a reduction in the expression of pro-inflammatory genes in the course of UC [34,35]. SCFAs may also alleviate intestinal inflammation by limiting excessive TLR receptor activity. This means that a diet rich in fibre can reduce the inflammatory responses associated with TLR4 [22]. Butyric acid also helps reduce damage to the intestinal barrier and regulate autophagy in intestinal epithelial cells [34]. A study showed that the vitamin D receptor (VDR) acts as a direct transcriptional regulator of ATG16L1 in intestinal epithelial cells, determining the efficiency of the autophagy pathway. VDR deficiency can lead to reduced expression of the ATG16L1 gene, Paneth cell dysfunction, intestinal dysbiosis, and progression of mucosal inflammation. Butyrate, produced by fibre fermentation, increases VDR expression, thus alleviating inflammation and supporting the VDR-ATG16L1 axis, directly improving intestinal barrier function [36]. Polyphenols, found primarily in plant products (fruit, green leafy vegetables, nuts, seeds, green tea, onions, and garlic), not only have anti-inflammatory properties but can also regulate signalling pathways associated with the immune response and inflammatory processes [30,33]. Substances such as curcumin, resveratrol, and epigallocatechin gallate have a regulatory effect on inflammatory mechanisms in IBD. Polyphenols can inhibit, among other things, NF-κB pathways dependent on TLR signalling pathways, which limits the production of cytokines and other inflammatory mediators and regulates the activity of immune system cells [30,37]. Additionally, Panaro et al. demonstrated in their study that curcumin limits TLR4 dimerisation, which inhibits signal transduction and cytokine production [38]. In turn, Wei et al. observed that curcumin can reduce the activity of the IL-23-Th17 axis, which also limits the formation of pro-inflammatory cytokines [39]. These studies show that polyphenols may be a promising method for alleviating chronic inflammation in IBD, but more research is needed to better understand their mechanisms of action [30,37,40].

A summary of genes associated with IBD is presented in Table 1.

Recent findings also suggest that the nutrigenomic effects of dietary components are closely linked with epigenetic regulation. These interactions between nutrients and the epigenome may provide novel targets for the prevention and treatment of IBD.

## 4. Epigenetic Mechanisms in Inflammatory Bowel Disease

Epigenetics is a field of biology concerned with the study of permanent or semi-permanent changes in gene expression that do not result from changes in DNA sequence but from chemical and structural modifications of chromatin. The most important of these include DNA methylation, histone modifications, the action of non-coding RNAs, and chromatin remodelling. These processes are strongly dependent on environmental factors, and some changes, such as methylation, can be permanent and pass on to subsequent generations of cells. Epigenetic mechanisms use a variety of strategies to regulate gene expression, which is crucial for proper development, differentiation, and function of the body. The integration of multiple research perspectives allows for a deeper understanding of the mechanisms underlying the development and treatment of IBD [41]. DNA methylation involves the attachment of methyl groups to cytosine within CpG dinucleotides. This process plays a key role in the regulation of gene promoter activity, affecting, among other things, inflammatory pathways and the functioning of the intestinal barrier [42]. These changes are dynamic and reversible, and their course depends on environmental factors, making them a potential target in the search for biomarkers and therapeutic strategies [43].

The deregulation of inflammatory gene promoters as a result of DNA methylation is one of the main pathogenic mechanisms of IBD. Methylation has been shown to affect genes crucial for the IL-23/IL-12 signalling axis, STAT3 and SOCS3 signalling axes, leading to disturbances in the regulation of the immune response and perpetuation of chronic inflammation. Of particular importance is the hypermethylation of the SOCS3 promoter, which limits its expression and, as a consequence, promotes excessive activation of cytokine pathways in CD [42]. The promoter of CDH1, which encodes E-cadherin, also shows increased methylation in the inflamed mucosa of the ileum in patients with CD, which is associated with epithelial barrier dysfunction and a potentially increased risk of developing colon cancer [44]. Additionally, methylation of promoters of genes responsible for apoptosis and regulation of immune responses, such as TCERG1L, indicates their involvement in both the aetiology of IBD and cancer processes [42].

CpG dinucleotides play a key role in transcription control and are one of the main areas of epigenetic modifications observed in IBD. In CD patients, different methylation patterns have been demonstrated of thousands of CpG sites between healthy and inflamed tissue. In addition, specific methylation profiles have been identified in a group of patients who underwent surgery and were treated conservatively, reflecting both the severity of the disease and its clinical course [45]. The study by Ventham et al. (TOPPIC) identified methylation differences at specific positions, including the WHSC1, EFNA3 and ITGB2 regions, which may be associated with recurrence after intestinal resection [46]. In addition, significantly increased methylation of selected CpG sites was found in the CDH1 gene—present in 90% of CD patients compared to 50% in the control group. These changes, limited to the intestinal mucosa and not present in peripheral blood, confirm the key importance of local epigenetic regulation in the maintenance and progression of the inflammatory process [44].

In recent years, DNA methylation has become a key area of research for diagnostic and prognostic biomarkers in IBD [43]. Changes in genes that regulate the inflammatory response, such as SOCS3 and ZBTB7B, are indicated as potential markers of disease progression [42]. Epigenome analyses have also allowed the identification of predictive signatures associated with the risk of surgical intervention and the escalation of biological treatment. “Epigenetic clocks of methylation” originally used in ageing studies, have also been shown to be a marker of rapid immune ageing in patients with CD, which is correlated with disease recurrence after resection [46]. Methylation at the AHRR locus (cg05575921), a biomarker of tobacco smoke exposure, also plays a role in differentiating the profiles of IBD patients. Thus, methylation biomarkers can not only reflect pathogenesis but also support personalisation of therapy [47].

DNA methylation patterns are strongly modulated by diet and gut microbiota. A Western diet, rich in fats and sugars and low in fibre, increases the risk of IBD [44]. Dietary components such as folic acid, vitamin B12, and polyphenols modulate the activity of DNA methyltransferase enzymes, affecting the methylation status of genes that regulate the inflammatory response. The Mediterranean diet is associated with beneficial changes in the epigenome, improved immune function, and reduced oxidative stress [48]. In addition, the gut microbiota, through the production of SCFAs [43]. Epigenetic histone modifications play an important role in regulating the activity of genes controlling the immune response in IBD [22,49].

Butyric acid limits neutrophil functions, including their pro-inflammatory activity, which is associated with the alleviation of intestinal mucosal inflammation [49]. This mechanism is the result of HDAC inhibition and regulation of the expression of genes responsible for the inflammatory response. These observations have been confirmed in clinical trials—butyrate supplementation in patients with active UC resulted in a reduction in disease severity, lower levels of inflammatory markers, and improved psychological parameters [50]. Molecular mechanisms also include the involvement of HDAC3 in intestinal epithelial cells, which regulates the expression of class II MHC and the balance between Th17 and Treg responses. Disruption of this pathway leads to an exacerbation of the inflammatory response, while proper modulation of HDAC3 by SCFA promotes immune tolerance to the intestinal microbiota [51]. Furthermore, SCFAs activate the p38 MAPK by inhibiting HDAC activity, which promotes the generation of B10 cells, characterised by anti-inflammatory properties due to the production of IL-10 [52]. Another important aspect is the role of propionate. This metabolite has been shown to inhibit HDAC activity and reduce the expression of pro-inflammatory cytokines, including IL-17, which is a key component of the Th17 response in IBD [53]. This mechanism is particularly important because Th17 lymphocytes are considered one of the main populations driving chronic inflammation in CD and UC. More broadly, SCFAs modulate histone acetylation in various types of immune cells, affecting both proliferation, differentiation, and the effector activity of the immune system [50,54].

One of the important epigenetic mechanisms in the pathogenesis of IBD is histone methylation regulated by EZH2. It is a catalytic subunit of the PRC2 complex responsible for the trimethylation of H3K27me3. The excessive activity of this enzyme promotes the intensification of inflammatory processes and disrupts the immune balance in the intestine. Studies have shown that the flavonoid lonicerin inhibits EZH2, enhancing autophagy and leading to inactivation of the NLRP3 inflammasome. The result is a reduction in the pro-inflammatory response [55]. EZH2 has also been shown to enhance intestinal epithelial cell apoptosis by inhibiting the activity of the JAK2/STAT pathway. Blocking this process results in reduced inflammatory activity and enhanced mucosal barrier function. This highlights the key role of EZH2 in the regulation of intestinal homeostasis [56]. In addition, EZH2 interacts in a complex with other PRC2 proteins, including EED. Inhibition of EED leads to a reduction in H3K27 methylation levels, thus limiting dendritic cell migration in a WNT pathway-dependent mechanism. This indicates a universal mechanism of action for PRC2, which may be relevant in the context of intestinal inflammation [57].

One of the epigenetic mechanisms in the pathogenesis of IBD is histone H3 trimethylation in lysine 4 (H3K4me3). It is responsible for regulating the activity of immune genes. Disruptions in H3K4me3 levels result in profound changes in the transcriptional programmes of immune and epithelial cells, which can exacerbate inflammatory processes [58]. Patients with IBD are characterised by diverse patterns of histone modifications within immune cells. In particular, changes in H3K4me3 levels were observed in NK cell populations, T lymphocytes, and CD34^+^ progenitor cells, which were correlated with disease activity [59]. Deregulation of this modification, as a marker of active promoters, promotes overexpression of pro-inflammatory genes and impaired immune tolerance. In CD, altered H3K4me3 patterns are observed in intestinal epithelial cells, which is correlated with the deregulation of cytokines and proteins responsible for mucosal homeostasis [60]. Excessive accumulation of H3K4me3 in the promoters of immune-related genes promotes the perpetuation of the inflammatory response [61]. In the UC model, the presence of H3K4me3 in the hypermethylated promoter of the NGF gene affects its expression, confirming the link between DNA methylation and histone modifications in the regulation of neuroimmunological processes in IBD [62].

For instance, altered expression of miR-21 and miR-146a has been associated with increased disease activity and disruption of epithelial barrier integrity [63,64]. Studies in animal models have shown that overexpression of miR-21 leads to increased intestinal epithelial permeability, promotes translocation of bacterial components, and modifies the expression profile of tight junction proteins, including various Claudin isoforms. Importantly, inhibition of miR-21 leads to improved intestinal barrier function and alleviation of inflammatory symptoms [63]. It has also been shown to have diagnostic potential as a non-invasive biomarker, allowing differentiation between disease entities within IBD [64].

The second important microRNA is miR-146a, which acts as an inflammatory response suppressor. Studies in mouse models have shown that miR-146a deficiency results in exacerbated colitis, while administration of its mimics silences broad networks of pro-inflammatory genes [65]. At the molecular level, miR-146a-5p regulates the response to interleukin 1β by inhibiting the IRAK1/TRAF6 pathway and, consequently, reducing the production of cytokines such as IL-6 and TNF-α [66]. Increased expression of the miR-143/145 cluster in colon epithelial cells has a protective effect, limiting the inflammatory process and reducing the risk of developing colon cancer associated with chronic inflammation [67]. In contrast, CD showed that hypermethylation of the miR-145 promoter region leads to its inhibition, affecting the regulation of the SOX9/CLDN8 signalling pathway and consequently affecting the integrity of the epithelial barrier [68]. The miR-148/152 family also plays an important role in the regulation of the intestinal barrier. In mice with a knockout of these miRNAs, increased expression of MMP10 and MMP13, activation of the NF-κB pathway, and consequently increased inflammation and a predisposition to developing colon cancer were observed [69]. This indicates their protective nature in terms of both the integrity of the intestinal epithelium and tumorigenesis processes [70]. Other important regulators include miR-195-5p, which inhibits CLDN2 expression in UC, thereby limiting excessive intestinal epithelial permeability [71]. In turn, miR-24 weakens the function of the intestinal barrier by interacting with the cingulin protein (CGN), which promotes increased intestinal permeability [72]. Exosomes rich in miR-223 inhibit the expression of CLDN8 in epithelial cells, leading to a weak intestinal barrier and increased susceptibility to pathogen penetration [73]. Many miRNAs—including miR-21, miR-146a, miR-143/145, and miR-148/152—act together in inflammatory processes and barrier integrity, modulating the immune response in IBD [74].

## 5. The Influence of Nutritional Components on Epigenetic Regulation in the Course of IBD

Nutrients can modulate gene expression and immune response not only directly but also through epigenetic mechanisms such as DNA methylation, histone modifications, and microRNA regulation. In the context of IBD, diet affects both the course of the disease and the effectiveness of therapy.

### 5.1. The Influence of Methyl Donors on DNA Methylation and the Risk of IBD

Methyl donor molecules play a key regulatory role in the epigenetics of the body, e.g., by participating in DNA methylation [75]. The body can obtain them exogenously from the diet and endogenously from methyloneogenesis [76]. Exogenous methyl donors include folates, vitamin B12, choline, and methionine. S-adenosylmethionine (SAM) is an endogenous methyl donor [76,77]. SAM synthesis occurs as a result of the coordinated action of methionine synthase (MS) and homocysteine betaine methyltransferase (BHMT) [76]. In MS, folic acid and vitamin B12 are cofactors involved in converting homocysteine back into methionine, which is essential for SAM production. In order for folic acid to participate in one-carbon metabolism as a cofactor, it must be converted to tetrahydrofolate (THF). THF is then converted to 5,10-methylenetetrahydrofolate, which is ultimately converted to L-5-methyltetrahydrofolate. In the next step, the methyl group of L-5-methyltetrahydrofolate is transferred to vitamin B12. Finally, the methyl group is transferred from vitamin B12 to homocysteine, converting it to methionine [77]. At the same time, mitochondrial oxidation of choline occurs in the presence of choline dehydrogenase (CHDH), resulting in the formation of betaine. The BHMT pathway uses it in the process of methionine resynthesis [76]. In the next step, SAM is synthesised from methionine and ATP in the presence of the enzyme methionine adenosyltransferase (MAT) [77]. It acts as a cofactor for DNA methyltransferase (DNMT) enzymes [78], which transfers methyl groups to cytosine residues in DNA [75]. After the methylation process is complete, SAM is transformed into S-adenosylhomocysteine (SAH), which is irreversibly converted to homocysteine in a reaction [76]. Disruption of homeostasis between SAM and SAH concentrations inhibits DNMT activity, affecting changes in DNA methylation [79]. DNA methylation profile abnormalities contribute to the development of IBD [80]. Patients with IBD often have deficiencies in folic acid and vitamin B12, which may be associated with the severity of their symptoms [75,77]. Insufficient intake of B vitamins, particularly B12, or excessive amounts of methionine in the diet, as well as homocysteine enzyme deficiencies, leads to hyperhomocysteinemia. Elevated homocysteine promotes inflammation, which in turn can increase the risk of IBD [81]. High concentrations of folates have both symptomatic relief and preventive effects in IBD [77]. Folate supplementation has been shown to prevent E. coli colonisation, which has a beneficial effect on neutralising inflammation in a mouse model of CD [75]. Dan et al. analysed the effect of dietary methyl donors on the likelihood of developing IBD associated with air pollution [82]. Conversely, deficiencies in methyl donors, such as vitamin B12, SAM, or choline, have been linked to exacerbation of colitis in animal models [83,84].

### 5.2. SCFAs as Natural Inhibitors of Histone Deacetylase

HDACs are enzymes responsible for the degradation of acetyl groups of histone proteins. They promote the formation of tightly packed chromatin and reduce gene activity. Inhibition of HDAC activity promotes the reduction in inflammation in the body, which has beneficial effects in the treatment of IBD [85]. The mechanisms of inhibition of HDAC by SCFAs have not yet been fully explained. SCFA is assumed to act indirectly by stimulating the G protein-coupled receptor (GPCR) or directly on HDAC [86]. The study by Ho et al. confirmed that butyric acid is the most effective HDAC3 inhibitor among SCFAs [87]. Butyric acid is the main inhibitor of HDAC enzymes of classes I, IIa, and IV [86]. However, no inhibitory effect has been demonstrated on HDACs6, HDAC10, and class 3 HDACs [88]. Butyric acid, by inhibiting HDAC9 activity, promotes histone H3 acetylation in the NOD2 promoter region [61] and H4 acetylation in the Tbx21 and Ifnγ genes [89]. SCFA affects immune system cells by inhibiting HDAC activity. It also reduces the expression of IL-6, IL-12, TNF-α, and nitric oxide (NO), and increases the expression of IL-22 [86]. In addition, it improves the affinity of hypoxia-inducible factor 1-alpha (HIF-1α) for the IL22 promoter. HDAC deactivation affects the forkhead box transcription factor P3 (Foxp3), which directs T cell differentiation to regulatory T cells (Tregs) releasing IL-10 [90]. HDAC inhibition reduces the expression of pro-inflammatory genes related to the STAT1 pathway, contributing to decreased intestinal inflammation [91]. Observations made by Caetano-Silva et al. confirm that a diet rich in soluble dietary fibre can increase the production of SCFAs, which in turn inhibited nuclear factor kappa B (NF-κB) and inflammation initiated by lipopolysaccharide (LPS) [92]. In addition, Feng et al. demonstrated that SCFAs, acting as HDAC inhibitors, suppress NLRP3 inflammasome activity, which promotes the maintenance of intestinal barrier homeostasis [93].

### 5.3. Modulation of Inflammatory Cytokines and MicroRNAs by Omega-3 and Omega-6 Fatty Acids

Omega-3 and omega-6 fatty acids are classified as polyunsaturated fatty acids (PUFAs), which are essential for the proper functioning of the organism [94,95]. Due to the inability of the human organism to synthesise PUFA, they must be supplied exogenously through diet. The recommended intake ratio of omega-6 to omega-3 fatty acids is 4:1 [94,96]. PUFAs shape inflammatory responses in the organism through the synthesis of lipid mediators, which regulate gene expression via microRNA (miR) [97]. By shaping miR activity, protein synthesis can be regulated, thereby determining the biological functions of the cell [98]. Omega-3 fatty acids such as EPA and DHA have anti-inflammatory effects based on the inhibition of pro-inflammatory genes that release cytokines and the modulation of eicosanoid synthesis [99]. EPA inhibits the synthesis of leukotriene B4 (LTB4), which is associated with a decrease in the expression of miR-146b and miR-125b-5p. It is also responsible for an increase in the number of miR-30a-3p, which is negatively correlated with the cellular Jun proto-oncogene (c-jun) and the cellular Fos proto-oncogene (c-fos). It also increases the amount of miR-30, which weakens the activity of Phosphoinositide 3-kinase adapter protein 1 (Pi3kap1) [100], while DHA is responsible for the increase in levels of let-7a, miR-21, miR-23b, miR-27b, and miR-320b [101]. EPA competes with AA for the active site of cyclooxygenase, which limits the formation of pro-inflammatory eicosanoids from AA. Omega-3 fatty acids activate the peroxisome proliferator-activated receptor (PPAR), the free fatty acid receptor 4 (FFA4), and the G protein-coupled receptor 40 (GPR40), which induces the synthesis of anti-inflammatory protectins and resolvins [102]. Resolvin increases the expression of miR-21, miR-146b, and miR-219, which inhibit the NF-kB signalling pathway [97,102]. This action results in a reduction in Monocyte Chemoattractant Protein-1 (MCP-1), Interleukin-1 beta (IL-1β), and TNF-α [99]. Furthermore, these acids reduce interleukin 8 (IL-8) and IL-6 expansion by deactivating the LPS-activated pathway [102]. In addition, they counteract the formation of Reactive Oxygen Species (ROS), which reduces the number of inflammasomes [99]. In addition, EPA and DHA increase the expression of anti-inflammatory cytokines such as IL-10 and interleukin-4 (IL-4) [102]. Flaxseed oil supplementation has been shown to reduce inflammatory markers in patients with UC [103]. On the other hand, omega-6 acids can have both pro-inflammatory and anti-inflammatory effects, depending on the type and ratio of omega-6 to omega-3 [104,105]. High LA concentration initiates the NF-kB pathway, which is positively correlated with the expression of TNF-α, IL-1β, IL-6 and IL-8 [104]. AA, via the PPARγ receptor, modulates the macrophage phenotype to the M2 type. This action leads to a reduction in LPS-induced inflammation and an increase in IL-10 expression. In addition, it limits the action of TLR4 signalling, leading to a reduction in TNFα and IL-6 levels [106]. In contrast, high levels of AA are metabolised to eicosanoids by cyclooxygenase (COX), lipoxygenase (LOX) and cytochrome P450 (CYP). The result of these actions is an increase in the activity of the NF-kB pathway and concentrations of TNF-α, IL-1β, and IL-6, as well as a decrease in IL-10 [107]. The AA metabolite lipoxin A4 (LXA4) induces miR-126-5p, which reduces NF-κB activation [108]. PUFA intake also affects the expression of various miRNAs associated with inflammation, suggesting a link between dietary fats, epigenetic regulation, and immune responses [109].

### 5.4. The Influence of Polyphenols on Epigenetic and Immunomodulatory Mechanisms

Curcumin is a lipophilic polyphenolic chemical compound extracted from the rhizomes of Curcuma longa L. Curcumin promotes the maintenance of immune homeostasis in the organism. It stimulates the differentiation of CD4^+^ T helper cells into Tregs in dendritic cells (DC), leading to the deactivation of antigen-specific T cells. It promotes the expression of type 2 helper T cells (Th2) while reducing the concentrations of type 1 helper T cells (Th1) concentrations. Limits the secretion of TNF-α and IL-6 through cytotoxic and inhibitory effects on B lymphocytes. It suppresses TLR4 expression, leading to decreased NF-κB activity and reduced production of pro-inflammatory cytokines, including IL-1, IL-6, and TNF-α [110]. By downregulating TLR4, it inhibits the differentiation of pro-inflammatory M1 macrophages [111]. Wei et al. investigated the therapeutic mechanisms of curcumin in colitis. In the study, colitis was induced in mice using sodium dextran sulphate (DSS), and then curcumin was administered orally for 7 days. After analysing the data, it was observed that curcumin shaped homeostasis was observed between Treg and type 17 helper T cells (Th17). Reduced IL-6, IL-17, and IL-23 concentrations and increased IL-10 levels in the colon [39]. In contrast, a clinical study conducted by Yu et al. included 75 healthy patients and 75 patients with UC. The effect of curcumin with chitosan on inflammatory markers was analysed. The data showed an increase in the expression of stromal cell-derived factor 1 (SDF-1), C-X-C chemokine receptor type 4 (CXCR4), miR-224-3p, and IFN-γ, and a decrease in TNF-α, TLR4, and NF-κB concentrations in the group taking curcumin with chitosan [112]. The modulating effect of curcumin on miR expression has also been demonstrated [113]. In vitro and in vivo studies in animal models demonstrate the ability of curcumin to attenuate miR-195-3p expression [114]. In addition, it may reduce the activity of miR-21 and miR-155 [111]. In addition, curcumin acts as an inhibitor of histone acetyltransferase p300 (HAT) through a proteasome-dependent degradation mechanism [115]. Inhibition of HAT activity minimises the acetylation of histone H3 in the IL-6 promoter, the expression of IL-6 mRNA, and IL-6 [113]. Curcumin also reduces HDAC1, 3, and 8 concentrations in Raji cells, which in turn increases histone H4 acetylation. Furthermore, curcumin has been shown to inhibit DNMT activity, resulting in hypomethylation [116].

Resveratrol is a phenolic chemical compound found in grapes, berries, and red fruits. It has a wide range of health benefits for the organism. Resveratrol inhibits the inflammatory response by modulating the signalling pathways TLR-4, TNF receptor-associated factor 6 (TRAF6), mitogen-activated protein kinase (MAPK) and protein kinase B (PKB/Akt) in LPS-stimulated macrophages. Promotes the cytotoxic activity of natural killer (NK) cells. It suppresses the activity of MHC class II molecules and CD80 and CD86, resulting in the arrest of DC phenotypic maturation and a reduction in T CD4^+^ levels. It is responsible for regulating B lymphocyte proliferation. It initiates the action of silent information regulator type 1 (SIRT1) leading to the blocking of p65/RelA acetylation, an element of NF-κB. These mechanisms contribute to the reduction in TNF-α, IL-6, COX-2, and matrix metalloproteinases (MMP)-1 and -3 levels [117]. Curcumin, resveratrol, and EGCG modulate immune and epigenetic pathways. Curcumin regulates T cell subsets and decreases TNF-α and IL-6 levels [118]. A study by Alrafas et al. demonstrates the anti-inflammatory effect of resveratrol, which consists of reducing the expression of miR-31, Let7a, and miR-132, which correlates with increased expression of Foxp3 [119]. In addition, resveratrol downregulates miR-17 and miR-520h and upregulates miR-34c, miR-663, miR-744, and miR-328 [120]. Increase SIRT1 expression, leading to histone deacetylation and reduction in CD40. In addition, it has the ability to inhibit certain HDAC families and lysine-1-specific demethylase (LSD1) involved in histone methylation regulation. It also reduces DNA methylation by inhibiting DNMT enzymatic activity and lowering 5-methylcytosine concentrations [121].

Epigallocatechin-3-gallate (EGCG) is a polyphenolic compound derived from green tea with beneficial health effects. EGCG inhibits LPS-activated DC maturation by blocking the MAPK and NF-κB pathways. It suppresses the expression of the NF-κB pathway activated by TLR2 and TLR4. It also reduces T lymphocyte expansion while regulating the TCD4^+^ to TCD8^+^ ratio. It weakens neutrophil transmigration through the endothelium and eliminates the expression of INF-γ, IL-2, IL-6 and TNF-α. In addition, it inhibits STAT1 activity, lowers TNF-α and IFNγ levels, and increases IL-4 concentrations. Maintains homeostasis between Th1 and Th2. Furthermore, by inhibiting mitochondrial DNA synthesis, EGCG suppresses the activity of the NLRP3 inflammasome in macrophages [122] Du et al. investigated the therapeutic effect of EGCG on DSS-induced IBD in mice. Following EGCG intervention, a reduction in IL-6, MCP-1 and TNF-α levels was observed, as well as inhibition of migration of CD3^+^ T cells and CD68^+^ macrophages [123]. EGCG binds directly to the enzymes DNMT1, DNMT3B, and HDAC1, blocking their activity, which results in a minimisation of DNA hypermethylation. By suppressing the activity of DNMT1, HDAC1, HDAC2, G9a, and the Polycomb repressive complex 2 (PRC2), EGCG promotes the interaction of histones H4 and H3K14 with the promoters of the C/EBPα, C/EBPε, p27 and CAF genes. It also stimulates the acetylation of histones H3 and H4 and reduces HAT activity [124]. EGCG affects multiple miRNAs involved in inflammation and epigenetic regulation [125,126].

### 5.5. The Effect of Vitamins on the Expression of Inflammatory Genes and Immune Cells

Vitamin D is a fat-soluble chemical compound classified as a steroid hormone [127]. The active form of vitamin D lowers the level of costimulatory molecules and class II MHC, which determines the expression of T lymphocytes. It suppresses the differentiation of immature B plasma cells and memory cells. It modulates downwards the production of IL-17, IFNγ, IL-21, IL-22, and IL-23, which influence the transformation of T helper cells into Th17 cells, as well as IL-12, IL-2, IFNγ, and TNF-α, which influence the transformation of T helper cells into Th1 cells. At the same time, it supports Th2 differentiation by upregulating IL-4, IL-5, and IL-10 in DCs and macrophages [128]. In addition, vitamin D promotes Treg cell differentiation and blocks cellular specialisation in Th17 via VDR/PLC-γ1/TGF-β1 signalling pathways. Vitamin D initiates macrophage polarisation toward the M2 type [129]. Studies on vitamin D in IBD show mixed results. Some report reductions in pro-inflammatory cytokines, such as TNF-α, IFN-γ, and IL12p70 [130], while others do not find significant changes in the expression of pro- or anti-inflammatory genes [131,132].

Vitamin E is a lipophilic chemical compound. It can alter the composition of the gut microbiota by increasing *Faecalibacterium* spp., *Lachnospira* spp., and thus indirectly affecting the lipid profile [133,134]. Vitamin E, as a powerful antioxidant, protects T lymphocytes from damage caused by oxidative stress. It stimulates the formation of synapses between TCD4^+^ and antigen-presenting cells. It strengthens the Th1 response and the function of neutrophils and NK cells, which deteriorate with age [135,136]. It improves macrophage function by reducing the production of prostaglandin PGE2. It stimulates IL-2 production by T lymphocytes. It also modulates the differentiation of T lymphocytes into Th1 or Th2. It also regulates DC maturation and migration [134]. However, by suppressing the NF-κB pathway, it inhibits DC maturation. In addition, it reduces the activity of IL-2, IL-17, IL-4, IL-6, IL-1β, and TNF-α [137]. A study by Saw et al. showed that the tocopherol-rich fraction (TRF) of vitamin E alleviates the symptoms of DSS-induced UC in mice. The researchers observed that TRF reduces inflammation by decreasing the expression of IL-6, IL-17, TNF-α, COX-2, and malondialdehyde (MDA) [138].

Effects of selected nutritional factors on epigenetic regulation, and immune function is presented in Table 2.

**Figure 1:**
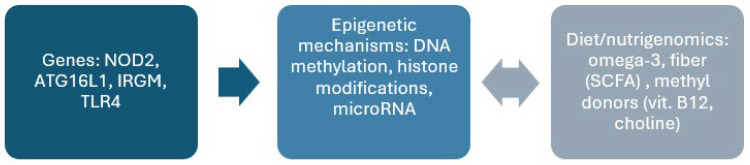
Summary of the interaction between genes, epigenetics, and diet in the pathogenesis of inflammatory bowel disease.

Inflammatory bowel disease results from a complex interaction of genetic, epigenetic, and environmental factors, including diet. Mutations in the NOD2, ATG16L1, IRGM, TLR4, and IL23R genes affect immune function and interactions with the gut microbiota. Epigenetic changes, including DNA methylation, histone modifications, and microRNA expression, can modulate immune responses. Selected dietary components play a key role in modulating gene expression and epigenetic mechanisms, e.g., omega-3 fatty acids, fibre, and methyl donors, leading to the maintenance of intestinal barrier homeostasis. Understanding the interrelationships between these mechanisms creates opportunities for the development of more precise therapeutic and preventive strategies in the treatment of inflammatory bowel diseases (Figure 1).

## 6. Future Perspectives

Research on nutrigenomics and epigenetics in IBD points to the growing potential of personalised nutritional strategies. A patient’s individual genetic and epigenetic profile may determine the effectiveness of a diet in modulating immune response, inflammation, and intestinal barrier function. A diet rich in omega-3 fatty acids, fibre, certain vitamins, and polyphenols has been shown to regulate the expression of pro- and anti-inflammatory genes, which may contribute to alleviating symptoms and reducing the risk of IBD exacerbation. In the future, it will be crucial to combine the nutrigenomic approach with the analysis of gut microbiota and epigenetic markers, which would enable the development of precise, individualised nutritional therapies for this group of patients.

## 7. Limitations

Many available studies are based on in vitro or animal models, which, although valuable for understanding molecular mechanisms, may not fully reflect the complex interactions that occur in humans. In addition, the studies analysed often include heterogeneous patient populations that differ in age, gender, disease phenotype (CD or UC), nutritional status, and treatment regimens, limiting the comparability and reproducibility of results. In addition, there are a limited number of clinical studies evaluating the direct impact of individual dietary components on specific epigenetic modifications in patients with IBD. Therefore, there is a need for multicentre, long-term studies combining genomic and epigenomic analyses to confirm the observed interactions in patients with IBD.

## 8. Conclusions

Integration of knowledge in the fields of nutrigenomics and epigenetics allows for a better understanding of how diet affects the expression of genes related to immunity and the functioning of the intestinal barrier. Including appropriate bioactive ingredients in the diet can modulate epigenetic processes, reduce the expression of pro-inflammatory genes, strengthen the intestinal barrier, and support the immune response. This approach offers promising opportunities for personalised nutritional therapy, both in the prevention and treatment of IBD.

## Figures and Tables

**Table 1:** Summary of genes associated with IBD.

Gene	Function	Functional Disorder
NOD2	Recognises bacterial pathogens, initiates autophagy [17]	Impaired immune response and increased susceptibility to inflammatory bowel disease [14,16]
ATG16L1 and IRGM	Crucial in autophagy, pathogen elimination [18,19,20]	Impaired intestinal homeostasis, increase in pro-inflammatory cytokines [18,19,20]
TLR4	Pathogen pattern recognition receptor [22]	Mucosal damage, decrease in SCFA, increase in inflammation [11,22]
IL23R	Regulates Th17 activity [16,18,19,23]	Chronic activation of the inflammatory response, intestinal fibrosis [16,18,19,23]

NOD2—Nucleotide-binding Oligomerisation Domain containing 2, ATG16L1—Autophagy Related 16 Like 1, IRGM—Immunity-Related GTPase M, TLR4—Toll-Like Receptor 4, IL23R—Interleukin 23 Receptor.

**Table 2:** Effects of Selected Nutritional Factors on Epigenetic Regulation, and Immune Function.

Nutritional Factor	Epigenetic/Immune Effect	Model/Population	References
Folates, vitamin B12	Regulation of DNA methylation; deficiencies can lead to hypermethylation and hyperhomocysteinemia	In vitro and animal	[75,77]
SCFA	HDAC inhibition, inhibition of CXCL10 release, reduction of pro-inflammatory cytokines. Improved intestinal homeostasis, reduced inflammation	In vitro, animal and human	[85,90,91,93]
Omega-3 (EPA, DHA)	NF-κB inhibition, microRNA modulation, increased IL-10	Animal and in vitro	[97,100,106]
Omega-6 (AA, LA)	Pro- and anti-inflammatory, proportion-dependent	In vitro	[105,106]
Curcumin, resveratrol, EGCG	NF-κB, STAT1, HDAC, inhibition; microRNA regulation, immune modulation	Animal, in vitro and human	[39,112,114,118,119,123,125]
Vitamin D	Inhibits Th1 responses, reduction in TNF-α levels, modulates cytokines	Human	[130,131,132]
Vitamin E	Reduction in pro-inflammatory cytokines, improvement in immune response, beneficial gut bacteria (*Faecalibacterium*, *Lachnospira*)	Animal and in vitro	[133,136,138]

DNA—Deoxyribonucleic Acid; SCFA—Short chain fatty acids; HDAC—Histone Deacetylase; IL-10—Interleukin 10; NF-κB—Nuclear Factor kappa B; AA—Arachidonic Acid; LA—Linoleic Acid; STAT1—Signal Transducer and Activator of Transcription 1; Th1—T Helper 1 Cells; EGCG—Epigallocatechin-3-gallate.

## Data Availability

No new data were created or analysed in this study. Data sharing is not applicable to this article.
